# Effectual Endeavors of Silk Protein Sericin against Isoproterenol Induced Cardiac Toxicity and Hypertrophy in Wistar Rats

**DOI:** 10.3390/life12071063

**Published:** 2022-07-15

**Authors:** Farogh Ahsan, Tarique Mahmood, Tanveer A. Wani, Seema Zargar, Mohammed Haris Siddiqui, Shazia Usmani, Arshiya Shamim, Muhammad Wahajuddin

**Affiliations:** 1 Department of Pharmacy, Integral University, Dasauli, Kursi Road, Lucknow 226026, India; faroghlab@gmail.com (F.A.); shazia@iul.ac.in (S.U.); arshiyanadeemsiddiqui@gmail.com (A.S.); 2 Department of Pharmaceutical Chemistry, College of Pharmacy, King Saud University, P.O. Box 2457, Riyadh 11451, Saudi Arabia; twani@ksu.edu.sa; 3 Department of Biochemistry, College of Science, King Saud University, P.O. Box 22452, Riyadh 11451, Saudi Arabia; szargar@ksu.edu.sa; 4 Department of Bioengineering, Integral University, Dasauli, Kursi Road, Lucknow 226026, India; mohdharis.siddiqui@gmail.com; 5 Institute of Cancer Therapeutics, School of Pharmacy and Medical Sciences, Faculty of Life Sciences, University of Bradford, Richmond Road, Bradford BD7 1DP, UK; m.wahajuddin@bradford.ac.uk

**Keywords:** sericin, myocardial infarction, silkworm, necrosis, hypertrophy, isoproterenol

## Abstract

The silkworm cocoon has been used in the treatment of various ailments in different Asian countries. This research was designed to evaluate the effect of sericin on myocardial necrosis and hypertrophy in isoproterenol-challenged rats. The rats were administered with sericin (500 and 1000 mg/kg, p.o.) for 28 days, followed by administration of isoprenaline (85 mg/kg, s.c.) on the 29th and 30th days. The cardioprotective activity was assessed by various physical, enzymatic, and histopathological parameters along with apoptotic marker expression. The cardioprotective effect showed that pre-treatment of rats with sericin significantly increased the non-enzymatic antioxidants marker in serum and heart tissue (glutathione, vitamin E, and vitamin C). The results were the same in enzymatic antioxidant marker, mitochondrial enzymes, and protein. The grading of heart, heart/body weight ratio, gross morphology, cardiac markers, oxidative stress markers in serum and heart tissue, glucose, serum lipid profiling and Lysosomal hydrolases, heart apoptotic markers such as MHC expression by western blot, apoptosis by flow cytometry, total myocardial collagen content, fibrosis estimation, myocyte size were significantly decreased when compared with isoproterenol (ISG) group however histopathological studies showed normal architecture of heart in both control and treated rats. The pharmacological study reflects that sericin on both doses i.e., 500 mg/kg and 1000 mg/kg have potent cardioprotective action against the experimental model which was confirmed by various physical, biochemical, and histopathological parameters evaluated further research is required to examine the molecular mechanism of cardioprotective effect of sericin.

## 1. Introduction

Among many, cardiovascular diseases (CVD) are one of the leading causes of death worldwide. According to the 2021 statistical data, globally, almost 18.6 million people died due to cardiovascular ailments in 2019, the current year for which global statistics are calculated. It displayed a 17.1% increase over the last decade. More than 523.2 million incidences of cardiovascular ailments in 2019 were recorded, a 26.6% increase when compared to 2010 [1]. The annual number of CVD deaths in India is expected to increase by 2.26 million from 1990 to 4.77 million in 2020 [2]. Coronary artery disease (CHD) incidence rates in India have been evaluated over the past few decades and ranged from 1% to 13.2% among urban residents and 1.6% to 7.4% among rural residents. Myocardial necrosis leads to the death of heart tissue from sudden lack of circulating blood, followed by hypertrophy leading to growth and enlargement of heart muscle cells [3]. Medicines of natural origin continue to show promising effects in developing countries. The current scenario of using natural remedies has created a great need for accurate and up-to-date information about the natural remedies’ properties, uses, efficacy, safety, and quality of medicinal plant products. Drug discovery needs to develop strong and potent lead molecules that, after a preliminary screening it must be successful in a medical candidate, and emerge through structural identification and elucidation through numerous new technologies. A prominent feature of naturally occurring products is their structural diversity, leading to their continued importance in drug discovery, which is still largely intact [4].

Ischemic heart disease (IHD) is the underlying cause of stroke, myocardial infarction, and kidney failure. Myocardial necrosis is a ubiquitous demonstration of ischemic heart disease. Cardiac necrosis is a situation which among the most worrying and frightening outcomes among IHD, Biochemical changes such as free radical damage, hyperlipidemia, lipid peroxidation, and hyperglycemia lead to quantitative and qualitative changes in the myocardium [5].

Cardiac hypertrophy is a compensatory mechanism for myocardial loss that occurs in combination with cardiovascular disease. An increase in collagen content reflects the occurrence of interstitial fibrosis, which has been observed in many cases of myocardial hypertrophy resulting from various forms of ventricular pressure overload. A change in cardiac structure eventually leads to changes in cardiac function that may be responsible for the development of congestive heart failure, ventricular arrhythmia, coronary artery disease, or sudden cardiac death [6].

Isoproterenol, produces highly cytotoxic free radicals which are used to stimulate peroxidation of membrane phospholipids leading to severe damage to the membrane of the myocardium resulting in an infarct-like necrosis of the cardiac tissue in experimental animal. ISO-induced myocardial necrosis establishes a well-standardized model for the reason that the pathophysiological changes in cardiac muscle of experimental animals are analogous to that perceived in human myocardial infarction or necrosis [7].

Silk sericin is an animal origin macromolecular protein resulting from *Bombyx mori* cocoon and constitutes 25–30% of silk protein. *Bombyx mori* cocoon comprise two types of protein named sericin and fibroin. Sericin contains predominantly amino acid groups like serine (40%), glycine (16%), glutamic acid, aspartic acid, threonine, and tyrosine. Sericin, has been explored for its numerous therapeutic potentials such as antioxidant activity associated with low solubility of sericin which magnifies its use in the medical field, such as anticancerous, antimicrobial, anticoagulant and anti-inflammatory, wound healing, and improving lipid profile [8].

*Bombyx mori* (Abresham) extract is the first drug mentioned in Avicenna’s tract of cardiac drugs, which is a collection of 64 cardioprotective drugs obtained from natural sources and it is also the chief constituent of a famous unani formulation viz. Khamira Abresham which is used in the treatment of cardiac ailment since ancient times [9]. The sericin is the pharmacologically active component of *Bombyx mori*. So, it is worthwhile to explore the role of sericin alone as a natural drug in the treatment of cardiovascular ailments especially myocardial necrosis and cardiac hypertrophy. Thus, this study is designed to evaluate the cardioprotective effect of sericin against isoproterenol-induced myocardial necrosis and hypertrophy in rats.

## 2. Material and Method

### 2.1. Chemicals

The test substance sericin, Aldrich now Sigma Merck (Burlington, MA, USA) (SKU: S5201). Other reagents/chemicals/diagnostic kits used in this study were bought from a local chemical supplier and were of research/analytical grades.

### 2.2. Animals

The specific pathogen-free (SPF) wistar rat (4-weeks-old) (150–180 g) of both sexes were used in this pharmacological study. These were acquired from the animal house center of the central drug research institute (CDRI), Lucknow. During the investigation time frame, the rats were lodged in a room in which the temperature was kept around 23 ± 2 °C with relative humidity of 50 ± 20%, in the presence of artificial lighting for 24/7 at 150 to 300 Lux of light intensity and air was changed for 10 to 15 times in every hour. The rats were acclimatized as mentioned above to research facility conditions for 7 days before initiation of the study. Not more than 3 rats were accommodated in polypropylene made cages (240 × 330 × 210 mm) during the acclimatization phase and 1 rat per cage during the dosing time frames and later this protocol was followed for the entire research period. A nomenclature was performed for all different experimental groups and the codes were written on the cages. Animals were fed with rodent specific pellet diet (Dayal animal feed) and normal drinking water *ad libitum* during this study [7].

### 2.3. Ethical Approval

The entire experiment was conducted according to the guidelines of CPCSEA. The research protocol was affirmed by Institutional Animal Ethical Committee (IAEC) with approval number (IU/IAEC/17/01), (Reg no.1213/PO/Re/S/08/CPCSEA, 5 June 2008) of faculty of pharmacy, Integral University, Lucknow (U.P.) India.

### 2.4. Treatment Protocol

#### 2.4.1. Dose Determination and Its Administration

Rats were weighed (150–180 g) and were arbitrarily distributed into 6 groups with six rats in each group (*n* = 6) as mentioned below. They were marked for individual identification and housed in their respective cages for 7 days before dosing to consider acclimatization to the research facility conditions. Sericin (500 and 1000 mg/kg b.w.) was dissolved in water and administered per orally (p.o.) by intubation method once a day for 28 days. Rats were treated according to treatment protocol orally by the aid of oral fan eeding needle daily for 28 days. On the 29th day, myocardial damage was incited in experimental rats by administration of isoproterenol (85 mg/kg/day, s.c.) twice at an interim of 24 h (i.e., given on the 29th and 30th days of treatment) while normal control and *per se* group rats were given an equivalent volume of the vehicle on 29th and 30th day [10]. The treatment protocol has been mentioned in Table 1.

By using the cervical dislocation method, the animals were sacrificed on the 31st day. Non-heparinized and heparinized centrifuge tubes were used for the collection of blood sample, while the rat’s heart was removed and later stored in formalin.

#### 2.4.2. Processing of Tissue and Blood Samples

##### Serum Preparation

Blood was gathered in a dry test tube and was permitted to coagulate at room temperature for around 40 min. Serum was isolated by centrifugation at around 5000 rpm for 10 min. After centrifugation, serum was collected carefully by micropipette without disturbing any cellular component of the tube. The serum samples were used for cardiac markers assay.

##### Plasma Preparation

The blood, gathered in a heparinized centrifuge tube, was centrifuged for 10 min at 4000 rpm and the plasma was separated which was removed by aspiration. The temperature of plasma should be maintained up to 2–8 °C during handling. The fresh separated plasma was utilized for the evaluation of various biochemical parameters, the remaining were divided into 0.5 mL aliquots and were stored at −20 °C [11].

##### Tissue Homogenate Preparation

Heart tissues (auricle and ventricle) (200 mg) were chopped into pieces maintaining the cold chain, and were homogenized in a specific buffer in ice-cold condition (pH 7.0) to result about 10% homogenate (*w*/*v*). The obtained homogenate was subjected to centrifugation at 12,000 rpm at 4 °C for 10 min in ice-cold centrifuge. The supernatant was separated and collected carefully without disturbing the tissue pellet. It was used for the various apoptotic or necrotic marker estimation. The sample was immediately used for analysis while the remaining was frozen immediately and was stored in a −80° freezer.

### 2.5. Parameter Estimated

#### 2.5.1. Grading of Heart

The hearts were dissected, washed out with Ice-cold saline solution blotted with filter paper, then measured, and photographed for heart grading. The cardio morphology was evaluated such as hearts for inflammation, redness, capillary dilatation, development of lesions, color, in all parts of the heart, and grading (Table 2) [12,13].

#### 2.5.2. Heart:Body Weight Ratio

The animals were euthanized, weighed and total body weight was recorded. Rats were placed on their back and pinned with extended extremities (inner hands and foot side) on board. To get rid of hair and dander, the mouse was washed or wetted with 70 percent ethanol. Dissection of the aortic root immediately above the aortic valves and the superior vena cava above the atria was performed followed by removal of the heart. With the help of forceps, mediastinal fat pads were carefully removed from the excised heart. Blood from the heart was drained by rubbing the heart on the surface of Kim wipe (through absorbent pad) or surgical compress. This cycle was repeated until it completely dries the heart. The dried heart was weighed and recorded later the heart was placed ina fixative position [14].

#### 2.5.3. Gross Morphology

For the evaluation of left ventricular wall and right ventricular free wall thicknesses (LV and RV), interventricular septum (IVS) portions of formalin stored or immediately excised hearts were manually cut. A camera (Nikon, Tokyo, Japan) was used for taking digital images of the section of each treatment group along with a metric scale. Morphometry of cross-sectional heart images was carried out using NIH Image J technology (NIH, Bethesda, MD, USA) (Version. 1.8.0_112) [15].

#### 2.5.4. Cardiac Markers

Later following parameters were analyzed from the stored serum: serum glutamic oxaloacetic transaminase (SGOT) or aspartate transaminase (AST), serum glutamic-pyruvic transaminase (SGPT) or alanine aminotransferase (ALT), alkaline phosphatase (ALP), lactate dehydrogenase (LDH) by using a commercially available diagnostic kit (Coral clinical system, Goa, India), creatine kinase (CK) and Creatine kinase- myocardial band (CK-MB) by using diagnostic kit manufactured by Agappe diagnostic, Kochi, India.

#### 2.5.5. Troponin

Troponin-I is the gold marker of myocardial infarction. The test is based upon the immuno-chromatographic principle. The estimation was performed by using a commercially available diagnostic kit (OSCAR, Troponin Kit, New Delhi, India).

#### 2.5.6. Oxidative Stress Parameter

Thiobarbituric acid (TBA) assay (TBARS) (serum and tissue) estimated malondialdehyde by [16,17] respectively. Lipid hydroperoxide (HP) in the plasma/tissues was assessed by the estimation method of [18]. Conjugated dienes (CD) were examined by the technique depicted by [19].

#### 2.5.7. Antioxidant Parameters

##### Non-Enzymatic Antioxidants Marker (Serum/Plasma)

Estimation of ascorbic acid was performed by the method of [20]. Colorimetric method was used for the estimation of serum vitamin E depicted by [21]. Reduced glutathione (GSH) was assessed by the procedure of [22].

##### Non-Enzymatic Antioxidants Marker (Heart)

Ascorbic acid was assessed by the help of titration strategy depicted by [23]. It was estimated by the method depicted by [24]. The estimation of reduced glutathione was performed by [25].

#### 2.5.8. Enzymatic Antioxidant Marker (Heart)

The following enzymatic antioxidant markers such as superoxide dismutase (SOD) [26], Catalase (CAT) [27], glutathione peroxidase (GPx) [28], glutathione reductase (GR) [29], glutathione-s-transferase (GST) [30] were estimated.

#### 2.5.9. Protein Estimation

The protein was estimated by biuret method described by [31]. The AGR (albumin:globulin ratio) was determined utilizing the formula

$$\mathrm{AGR}=\mathrm{albumin}/\left(\mathrm{total}\;\mathrm{protein}-\mathrm{albumin}\right)$$

#### 2.5.10. Estimation of Glucose

The estimation was performed by the commercially available kits of Excel diagnostics Pvt. Ltd., Hyderabad, India) (GOD/POD Method; Code: 11018/19/20).

#### 2.5.11. Lipid Profile

##### Estimation of Serum Lipids

The level of serum total cholesterol, plasma triglycerides (TG), low density lipoprotein (LDL), high-density lipoprotein (HDL), very low-density lipoprotein (VLDL), free fatty acid (FFA), phospholipids were evaluated by the enzymatic technique depicted by [32,33,34,35,36,37] respectively.

##### Estimation of Tissue Lipids

Extraction of tissue lipids was performed by the methods of [38]. The level of tissue total cholesterol, triglycerides (TG), free fatty acids (FFA), and phospholipids were estimated by the methods of [32,33,36,37] respectively.

#### 2.5.12. Lysosomal hydrolases

The effect of β-glucosidase, and β-galactosidase were tested by the strategy depicted by [39]. The assay of α-galactosidase, Cathepsin B, Cathepsin-D was performed by the methods of [40,41,42] respectively.

#### 2.5.13. Heart Mitochondrial Enzymes

The heart mitochondrial portions were separated by the modified methods of [43]. Isocitrate dehydrogenase (IDH), malate dehydrogenase (MDH), α-ketoglutarate dehydrogenase (KDH) and succinate dehydrogenase (SDH) were estimated by the method of [44,45,46,47] respectively.

#### 2.5.14. Apoptotic Marker

##### α- and β-Myosin Heavy Chain (α, β-MHC) Expression

Ventricular sample (*50 mg) from heart tissue, frozen at −80 °C was homogenized with ice-cold radioimmunoprecipitation assay buffer (RIPA buffer) (600 µL) (20 mM Tris-HCl (pH 7.5), 150 mM NaCl, 1 mM EGTA, 1 mM Na_2_EDTA, 1% NP-40, 1% sodium deoxycholate, 1 mM β-glycerophosphate, 2.5 mM sodium pyrophosphate, 1 mM Na_3_VO_4_ and1 µg/mL leupeptin) in Teflon-coated glass homogenizing tube with the help of polypropylene pestle at 4 °C. PMSF (6 µL) was added to the homogenate as PMSF inhibits the proteases which may be released at the time of lysis and can destroy the protein thus it inhibits any protein breakdown. The homogenate was passed through a 20-gauge needle and agitated for about 30 min at 4 °C. Homogenate was centrifuged at 12,000× *g* at 4 °C for a total of 15 min. After centrifugation, carefully supernatant was pipette out and stored at −20 °C and pellets were discarded. The supernatant was mixed with an equal amount of gel loading buffer [glycerol (20% *v*/*v*), Tris100 mM, pH 6.8; bromophenol blue (0.2% *w*/*v*); SDS (6% *w*/*v*) and beta-mercaptoethanol (200 mM)] and it was denatured by heating for about 3 min. SDS–PAGE was performed by loading the samples and running the gel (6%) at 100 V/30 mA at 4 °C. Transferred proteins to PVDF membrane (Millipore) at 25 V and a constant current of 1 mA/cm^3^ for 3 h. Blocked the membrane with 5% skim milk in TBS Tween (0.2%) for 2 h at room temperature. Incubated with primary antibody (1:1000) (Mouse anti-myosin heavy chain monoclonal antibody, R&D system, Minneapolis, MN, USA. #Cat num: MAB4470) in TBST overnight at 4 °C. Washed 3 × 15 min with TBST at room temperature. Incubated with secondary antibody (1:1000) (Polyclonal goat HRP-conjugated anti-mouse IgG secondary antibody, R&D system, USA, #Cat num: HAF007) for 2 h at room temperature. Washed 3 × 15 min with TBS Tween. Given a final wash with 1× TBS. β-actin was used as a loading control. The intensity of bands was detected by enhanced chemiluminescence (ECL) technique (Bio-Rad Laboratories, Hercules, CA, USA) and imaged by E-Gel Imager (Tanon-5200Multi, Shanghai, China). The bands obtained were selected from the scanned image of western blot followed by densitometry analysis for identical areas of bands was performed by using Image J software (version 1.51, NIH, Bethesda, MD, USA) [48,49].

##### Flow Cytometry for the Measurement of Myocardial Apoptosis

The myocardial apoptosis of rats was examined by flow cytometry/fluorescein isothiocyanate (FITC)-labeled annexin V. FITC-labeled annexin V is generally used as an extremely sensitive chemical to proportionately determine early apoptotic cells by calculating the percentage of apoptotically positive cells. Annexin V is a calcium-dependent phospholipid-binding protein that has a high affinity for binding to phosphatidylserine, which is present on the outer membrane of apoptotic cells. According to the protocol, myocardial tissues were attained from rats of various different groups later they were suspended in chilled 1× phosphate-buffered saline (PBS). Then the tissues were digested with collagenase and fibrinogen to obtain single cells. A total of 1 × 10^5^ cells were mixed with 300 µL of the binding buffer and the mixture was later incubated with 5 µL FITC-labeled annexin V (20 µg/mL) at room temperature in the dark for around 30 min, later it was incubated with 5 µL Propidium iodide (PI) (50 µg/mL) at room temperature in the dark for 5–15 min. The cells were then diluted using 400 µL binding buffer and flashed by FACScan flow cytometry to determine the percentage of apoptotic cells using FITC-labeled cells without annexin V and PI as serve as the negative control [50,51,52].

#### 2.5.15. Total Myocardial Collagen Content (Heart)

Hydroxyproline content in tissue samples and collagen extracts was estimated by the method depicted by [53].

The total collagen content of samples in g/100 g dry weight was estimated as follows, where,

Total collagen content (TCC): THP × CF

THP = Total hydroxyproline content (g/100 g dry weight)

CF = Conversion factor

The general conversion factor for hydroxyproline to collagen is 8.

#### 2.5.16. Fibrosis

The estimation was performed by the method of [54].

#### 2.5.17. Cell (Myocyte) Size Determination

The myocyte size was determined by the method depicted by [15]. Morphometry of left ventricular myocytes was performed in the H&E section of the lateral mid-free left ventricular wall. The cross-sectional area of myocyte was quantified from the myocytes that were transversely cut and possess both an intact cellular membrane and an evident nucleus. The outer boundaries of the myocytes were identified and the total myocyte areas were calculated. Approximately 100 myocytes, and cross-sectional areas were determined from each heart and were studied using the software named NIH Image J software. The size of the myocyte was expressed as µm^2^.

#### 2.5.18. Histopathology

Hearts were quickly removed after euthanasia, fats were removed carefully. washed with ice-cold saline, all clot was removed immediately and was fixed in 10% buffered formalin for 48 h, it was dehydrated by dipping successively in various different concentrations of water-ethanol, cleaned with help of xylene, again embedded in paraffin, and by the help of microtome 5–6 µm thick sections were cut. Sections were stained with haematoxylin and eosin dye. The specimens were evaluated with the help of a light compound microscope. The histopathological variations were analysed by a pathologist.

## 3. Results

### 3.1. Grading of Heart

**Figure 1:**
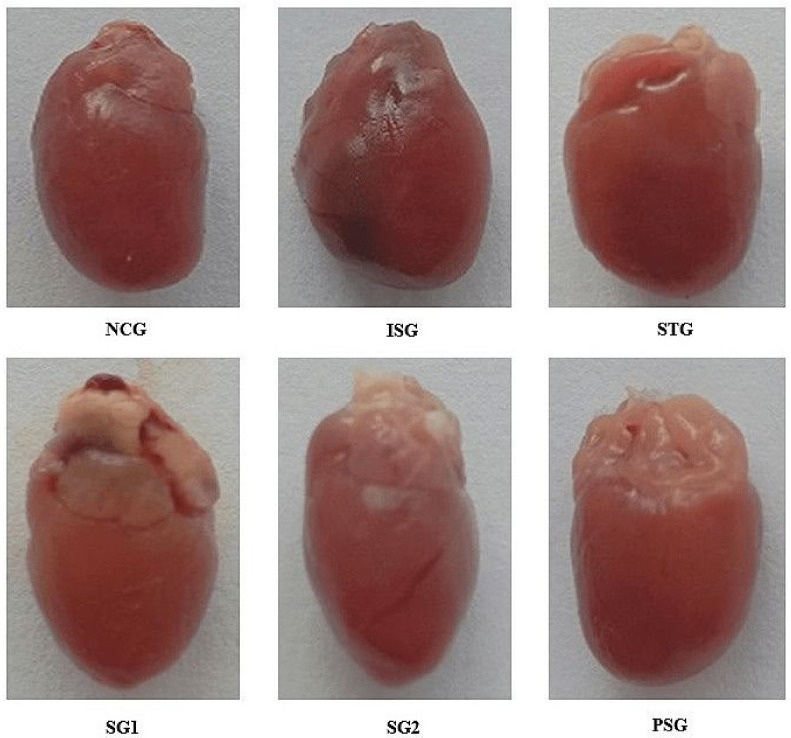
Visual pictograph of the heart showing morphological features in different treatment groups.

The visual examination of the heart provided the information related to the amount of necrosis in myocardial tissues (Figure 1).

Normal control group (NCG i.e., vehicle-treated) and *per se* group (PSG i.e., only sericin treated) showed no mark of necrosis or lesion (Grade-0). Isoproterenol control group (ISG i.e., Isoproterenol treated) showed scar formation, diffused heart, and an increase in necrosis was observed thus showing grade-5 category heart damage. Standard group (STG i.e., metoprolol treated) and sericin group 2 (SG2 i.e., sericin treated) showed a significant reduction in edema, dilation of capillary, and formation of scar thus showing grade-1 category cardiac damage. Sericin group 1 (SG1 i.e., sericin treated) showed slight edema and yellowing of cardiac fibers, but there was a reduction in scar formation showing the grade-2 category of cardiac damage (Table 3).

### 3.2. Heart:Body Weight Ratio

A mathematically calculated value in which the heart’s total weight is divided by the total body weight, and the result is shown as a ratio which is termed as the heart: body weight ratio.

**Figure 2:**
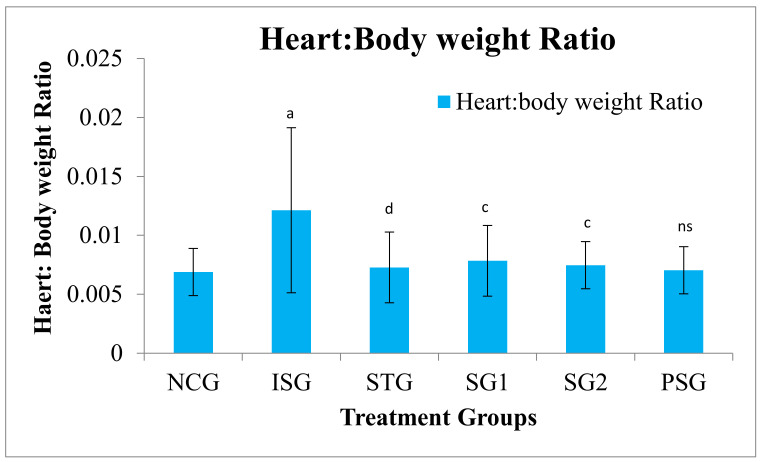
Heart:Body weight ratio of various treatment group. Where, values are expressed as mean ± SD; (*n* = 6) in each group. Data were subjected to one-way ANOVA followed by Dunnett’s test when STG, SG1, and SG2 groups were compared to the isoproterenol control group (ISG), while the isoproterenol control group (ISG) and *per se* (PSG) were compared to the normal control group (NCG), ^a^ *p* < 0.001, ^ns^ *p*> 0.05 when compared to the normal control group (NCG), ^c^ *p* < 0.01, ^d^ *p* < 0.001 when compared to the isoproterenol control group (ISG).

The heart:body weight ratio was statistically highly significant (*p* < 0.001) in isoproterenol group rats (ISG) when compared to (NCG). The sericin-treated group (SG1, SG2) showed a statistically very significant (*p* < 0.01) decrease in heart:body weight ratio when compared to the isoproterenol group (ISG) while the standard group (STG) showed a statistically highly significant (*p* < 0.001) decrease in heart:Body weight ratio when compared to isoproterenol group (ISG). The *per se* group (PSG) showed no significant (*p* > 0.05) change in terms of H:B ratio as compared with the normal control group (NCG) (Figure 2).

### 3.3. Gross Morphology

**Figure 3:**
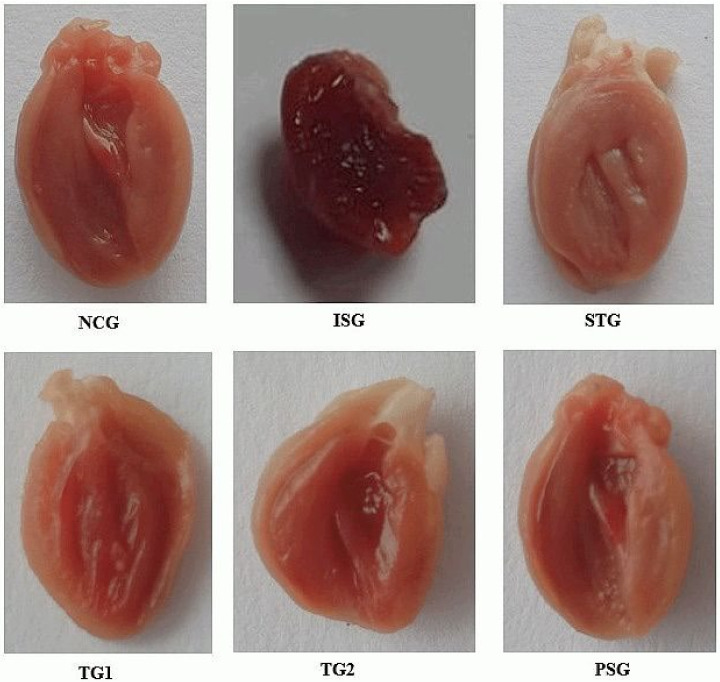
Gross morphology of heart displaying intravascular septum (IVS), right ventricular wall (RV), and left ventricular wall (LV) of different treatment groups.

**Figure 4:**
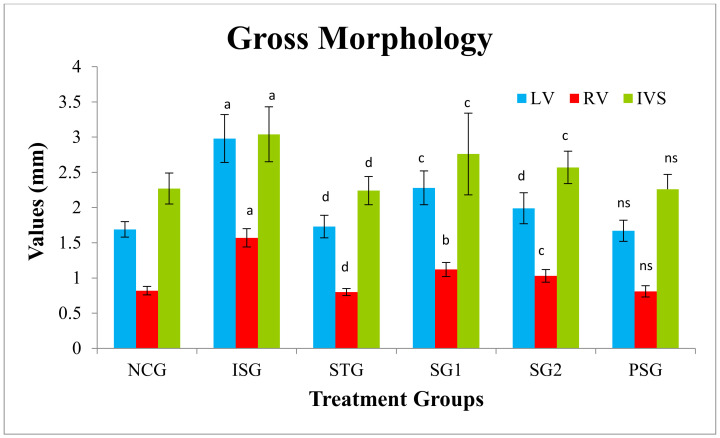
Gross morphology value of various treatment groups. Where, values are expressed as mean ± SD; (*n* = 6) in each group. LV: Left Ventricle, RV: Right Ventricle, IVS: Intravascular Septum. Data were subjected to one-way ANOVA followed by Dunnett’s test when STG, SG1, SG2 group were compared to isoproterenol control group (ISG), while isoproterenol control group (ISG) and *per se* (PSG) were compared to normal control group (NCG), ^a^ *p* < 0.001, ^ns^ *p* > 0.05 when compared to normal control group (NCG), ^b^ *p* < 0.05, ^c^ *p* < 0.01, ^d^ *p* < 0.001 when compared to isoproterenol control group (ISG).

The rat heart was cut down in a longitudinal fashion and was opened from between, later the RV, LV wall thickness, and IVS was measured (Figure 3). The intraventricular septum thickness (IVS), right ventricular wall thickness (RV), and left ventricular wall thickness (LV) showed a significant increase in ISG when compared to NCG. The sericin-treated group (SG1 and SG2) displayed a significant decrease in IVS, RV, LV when compared to the ISG group while the STG group showed a significant reduction in IVS, RV, LV when compared to the ISG group. PSG group displayed no significant differences in IVS, RV, and LV diameter when it was compared to the NCG group (Figure 4).

### 3.4. Serum Biochemical Markers

**Figure 5:**
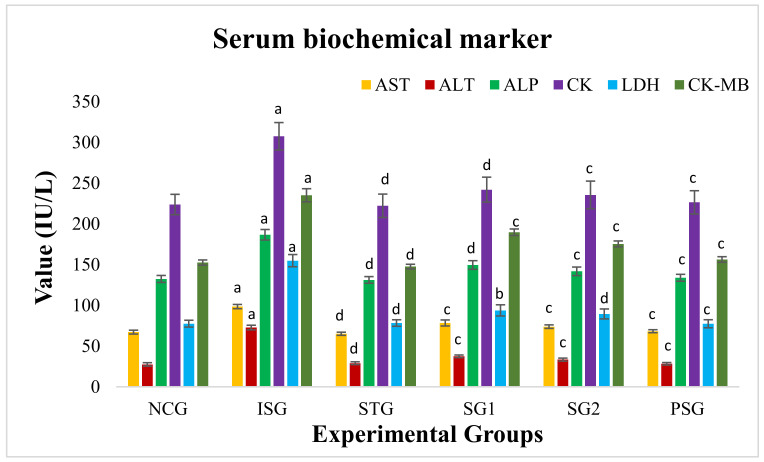
Serum biochemical estimation of various treatment groups. Where, values are expressed as mean ± SD; (*n* = 6) in each group. Aspartate aminotransferase, (AST) Alanine aminotransferase (ALT), Lactate dehydrogenase (LDH), Alkaline phosphatase (ALP), Creatine kinase (CK). Data were subjected to one-way ANOVA followed by Dunnett’s test when STG, SG1, SG2 group were compared to isoproterenol control group (ISG), while isoproterenol control group (ISG) and *per se* (PSG) were compared to normal control group (NCG), ^a^ *p* < 0.001, ^ns^ *p* > 0.05 when compared to normal control group (NCG), ^b^ *p* < 0.05, ^c^ *p* < 0.01, ^d^ *p* < 0.001 when compared to isoproterenol control group (ISG).

The activities of serum biochemical markers AST, ALT, ALP, CK, LDH, and CK-MB was found to increase in the ISG group when compared with NCG group rats. After 28 days of treatment with sericin the biochemical markers were found to be declined to near-normal levels. The outcome indicates clearly that the sericin prevented the myocardial tissue from enzymatic leakage from the cell sites, evidencing its protective effect on the myocardium. PSG group showed no significant differences (*p* > 0.05) in the level of AST, ALT, ALP, CK, LDH, and CK-MB when compared to the NCG group (Figure 5).

### 3.5. Troponin

**Figure 6:**
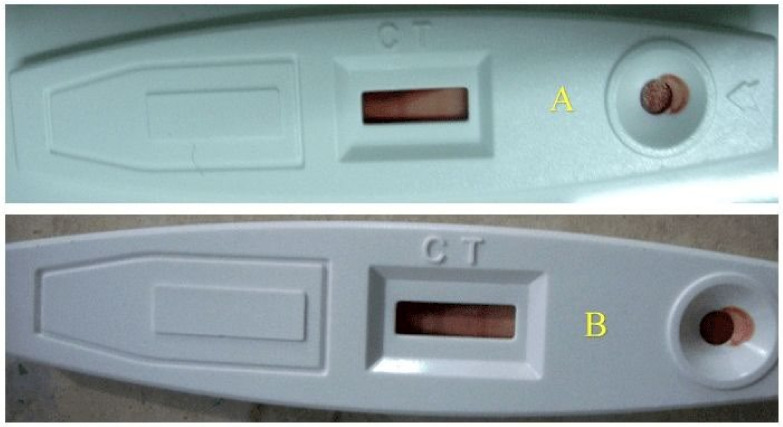
Troponin–I rapid test kit (**A**)—Single band indicates absence of marker; (**B**)—Double band indicates the presence of marker).

The result showed that all animals of isoproterenol treated group (ISG) showed the presence of troponin-I in each animal when it was compared to (NCG) while all animals of the standard group (STG) and *per se* group (PSG) showed an absence of troponin-I in their blood when compared to the normal control group (NCG), while three animals showed the presence of troponin-I in sericin group 1 (SG1), while only two animals showed the presence of troponin-I in sericin group 2 (SG2) when compared to isoproterenol treated group (ISG) (Table 4) (Figure 6).

### 3.6. Oxidative Stress Parameters

**Figure 7:**
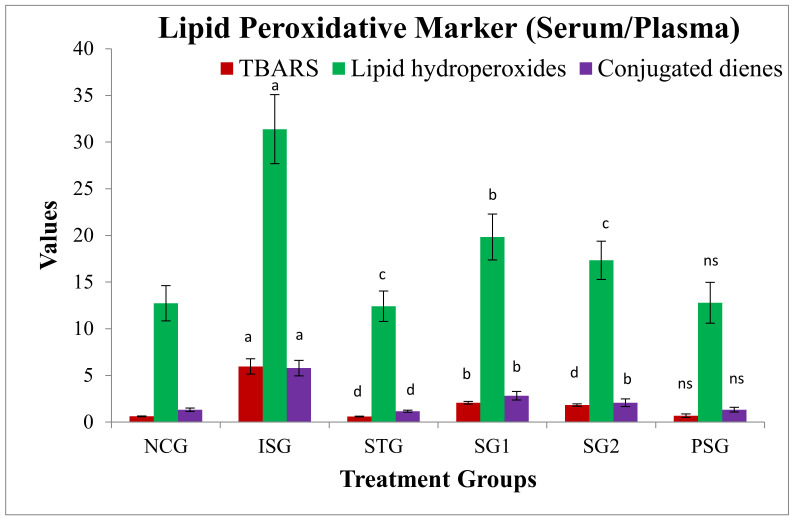
Lipid peroxidation markers level (serum/plasma) of various treatment groups. Where, values are expressed as mean ± SD; (*n* = 6) in each group. TBARS: Thiobarbituric acid reactive substances. Data were subjected to one-way ANOVA followed by Dunnett’s test when STG, SG1, and SG2 groups were compared to the isoproterenol control group (ISG), while the isoproterenol control group (ISG) and *per se* (PSG) were compared to the normal control group (NCG), ^a^ *p* <0.001, ^ns^ *p* >0.05 when compared to the normal control group (NCG), ^b^ *p* < 0.05, ^c^ *p* < 0.01, ^d^ *p* < 0.001 when compared to the isoproterenol control group (ISG). Where TBARS: Thiobarbituric acid reactive substances. MDA concentration in the serum is communicated as nmol/mL, Lipid hydroperoxides were reported as mmol/dL of plasma, and Concentration of CDs was expressed as mmol/dL of plasma.

**Figure 8:**
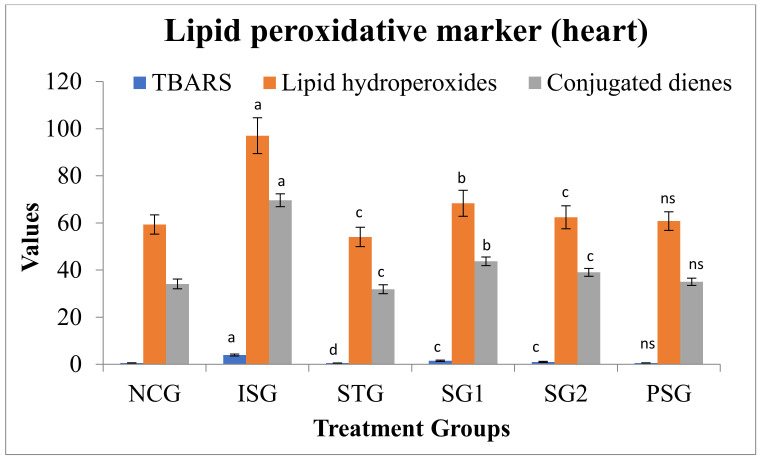
Lipid peroxidation markers level (heart) of different treatment groups. Where, values are expressed as mean ± SD; (*n* = 6) in each group. Data were subjected to one-way ANOVA followed by Dunnett’s test when STG, SG1, SG2 group were compared to isoproterenol control group (ISG), while isoproterenol control group (ISG) and *per se* (PSG) were compared to normal control group (NCG), ^a^ *p* < 0.001, ^ns^ *p* > 0.05 when compared to normal control group (NCG), ^b^ *p* < 0.05, ^c^ *p* < 0.01, ^d^ *p* < 0.001 when compared to isoproterenol control group (ISG). MDA concentration in the tissue was expressed as nmoles/mg; Lipid hydroperoxides were expressed as mmol/100 g of tissues; Concentration of CDs was expressed as mmol/100 g of tissues. TBARS: Thiobarbituric acid reactive substances.

A significant upsurge in the levels of lipid peroxidative marker in serum/plasma and cardiac tissue (TBARS, lipid hydroperoxides, conjugated dienes) in ISG group rats when compared to NCG was observed due to accumulation of free radicals. STG, SG1, and SG2 groups showed decreased levels of lipid peroxidative marker. The PSG group showed no significant difference in the level of lipid peroxidative marker when compared to the NCG group. The results indicate the high free radical scavenging property or strong neutralizing effect of sericin (Figure 7 and Figure 8).

### 3.7. Antioxidant Parameters

#### 3.7.1. Non-Enzymatic Antioxidants Marker in Serum/Plasma/Tissue

**Figure 9:**
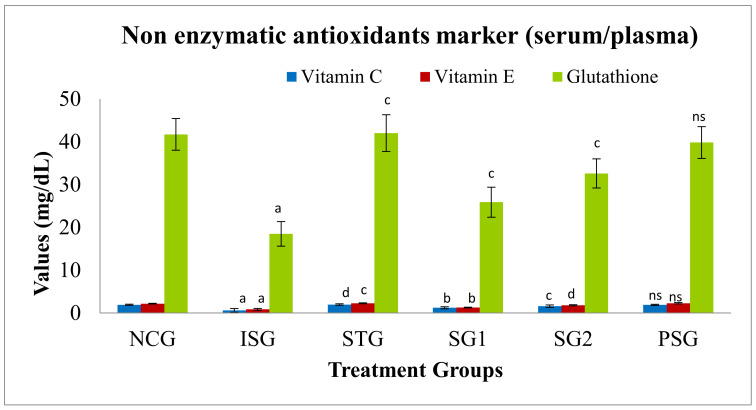
Non-enzymatic antioxidants marker level (serum/plasma) in different treatment groups. Where, values are expressed as mean ± SD; (*n* = 6) in each group. Data were subjected to one-way ANOVA followed by Dunnett’s test when STG, SG1, SG2 group were compared to isoproterenol control group (ISG), while isoproterenol control group (ISG) and *per se* (PSG) were compared to normal control group (NCG), ^a^ *p* < 0.001, ^ns^ *p* > 0.05 when compared to normal control group (NCG), ^b^ *p* < 0.05, ^c^ *p* < 0.01, ^d^ *p* < 0.001 when compared to isoproterenol control group (ISG).

**Figure 10:**
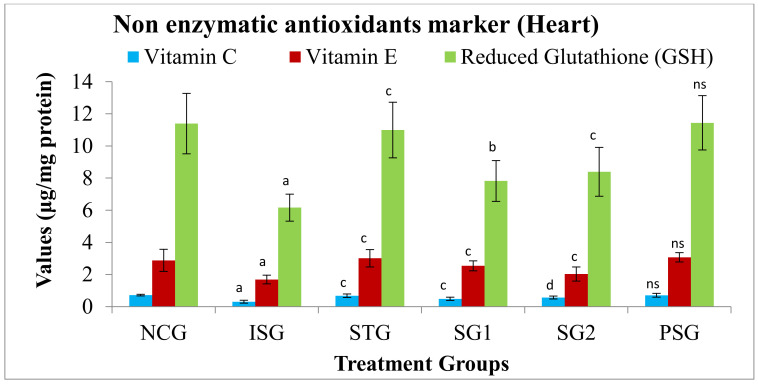
Antioxidants marker level (non-enzymatic) in the heart tissue. Where, values are expressed as mean ± SD; (*n* = 6) in each group. Data were subjected to one-way ANOVA followed by Dunnett’s test when STG, SG1, SG2 group were compared to isoproterenol control group (ISG), while isoproterenol control group (ISG) and *per se* (PSG) were compared to normal control group (NCG), ^a^ *p* < 0.001, ^ns^ *p* > 0.05 when compared to normal control group (NCG), ^b^ *p* < 0.05, ^c^ *p* < 0.01, ^d^ *p* < 0.001 when compared to isoproterenol control group (ISG).

The levels of non-enzymatic antioxidants (viz. vitamin C, vitamin E, and glutathione) were found to be decreased in the serum/plasma and myocardial tissue of groups intoxicated with ISO (ISG) when compared with the NCG group. The PSG group did not show any changes in antioxidant levels when compared to the NCG group. The STG, SG1, and SG2 groups, reversed the total amount of non-enzymatic antioxidants to near-normal levels in the serum/plasma and myocardial tissue thus proving that sericin possesses good anti-oxidant potential (Figure 9 and Figure 10).

#### 3.7.2. Antioxidant Marker (Enzymatic) in Heart

**Figure 11:**
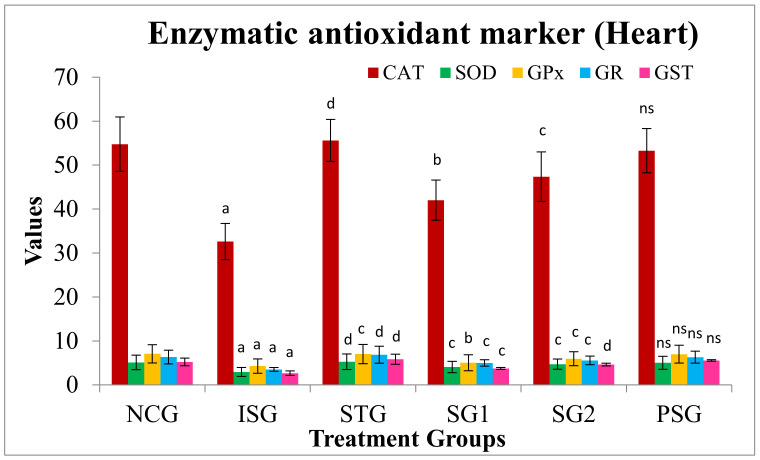
Enzymatic antioxidants marker level (heart) in different treatment groups. Where, values are expressed as mean ± SD; (*n* = 6) in each group. Data were subjected to one-way ANOVA followed by Dunnett’s test when STG, SG1, SG2 groups were compared to the isoproterenol control group (ISG), while the isoproterenol control group (ISG) and *per se* (PSG) were compared to the normal control group (NCG), ^a^ *p* < 0.001, ^ns^ *p* > 0.05 when compared to the normal control group (NCG), ^b^ *p* < 0.05, ^c^ *p* < 0.01, ^d^ *p* < 0.001 when compared to Isoproterenol control group (ISG). Catalase (CAT), Superoxide dismutase (SOD), Glutathione peroxidase (GPx), Glutathione reductase (GR), and Glutathione S-transferases (GSTs).

The amount of enzymatic antioxidants such as (GR), (CAT), (GPx), (SOD), and (GST) were decreased in ISO-induced rats (ISG) when compared with NCG rats. Pre-treatment of sericin and metoprolol for a period of 28 days restored the actions of these enzymes to near-normal levels in STD, SG1, and SG2 groups when compared to ISG group rats. The PSG group did not show any changes in antioxidant levels when compared to the NCG group (Figure 11).

**Units:** Glutathione peroxidase (GPx): μmole of GSH used/min/mg protein or μg of glutathione utilized/min/mg protein. Catalase (CAT): μmole of hydrogen peroxide disintegrated/min/mL; or μmoles of hydrogen peroxide used/min/mL. Glutathione reductase (GR): μg of condensed glutathione obtained/min/mg protein or μg of reduced glutathione utilised/min/mg protein. Glutathione-S-transferase (GST): μg of CDNB conjugate produced/min/mg protein or μg of CDNB conjugate utilised/min/mg protein *Superoxide dismutase* (SOD): The one unit of enzyme activity was reflected as the reaction of an enzyme that causes the inhibition of 50% of nitroblue tetrazolium (NBT) decrease/minute or the required concentration of enzyme that can inhibit the formation of chromogen by approximately 50% per min under optimal situation or SOD units/mg of protein.

### 3.8. Protein Estimation

**Figure 12:**
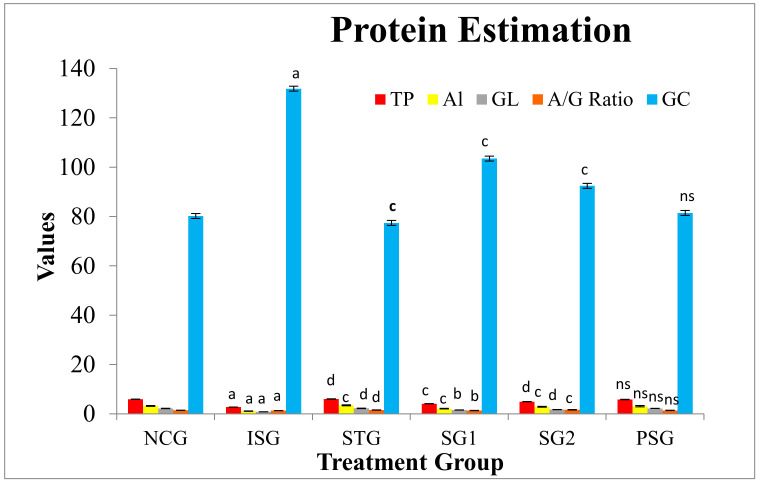
Total protein, albumin, globulin, and glucose level in the different treatment groups. Where, values are indicated as mean ± SD; (*n* = 6) in each group. Data were subjected to one-way ANOVA followed by Dunnett’s test when STG, SG1, SG2 group were compared to isoproterenol control group (ISG), while isoproterenol control group (ISG) and *per se* (PSG) were compared to normal control group (NCG), ^a^ *p* < 0.001, ^ns^ *p* > 0.05 when compared to normal control group (NCG), ^b^ *p* < 0.05, ^c^ *p* < 0.01, ^d^ *p* < 0.001 when compared to isoproterenol control group (ISG). Total protein (TP), albumin (AI), globulin (GL), albumin/globulin ratio (A/G Ratio), glucose (GC). Unit of TP, AL, GL was g/dL, while glucose was mg/dL.

There was a significant decrease in total protein (TP), globulin (GL), albumin (AL), and A/G ratio in ISO challenged rats (ISG). The outcome was reversed in the sericin and standard treated group (SG1, SG2, and STG), and the level of protein content rise to near normal. The result of glucose level was vice-versa when compared to protein content, In ISO challenged (ISG) rat, the glucose level showed elevation while reduction to near normal level was observed in sericin and standard treated group (SG1, SG2, and STG). PSG group did not show any significant changes in protein and glucose content. This finding confirms that sericin has potent protein synthesis ability and also takes the glucose level to near normal (Figure 12).

### 3.9. Lipid Profile

**Figure 13:**
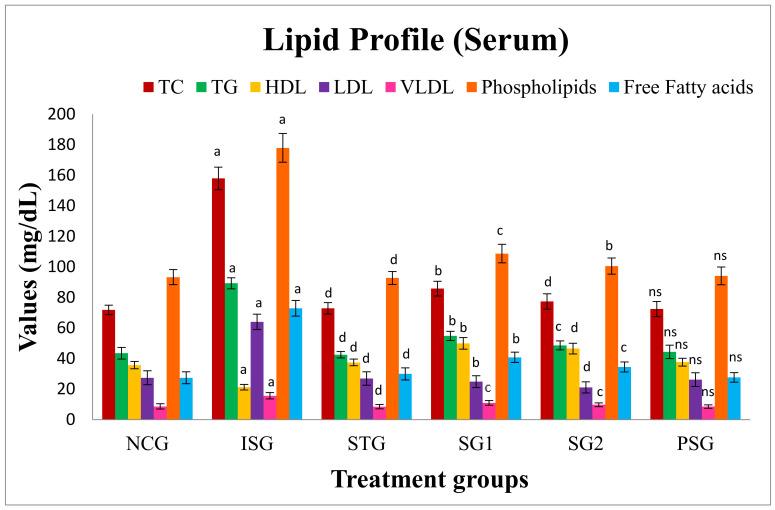
Lipid profile (serum) of different treatment groups. Where, values are indicated as mean ± SD; (*n* = 6) in each group. Data were subjected to one-way ANOVA followed by Dunnett’s test when STG, SG1, SG2 group were compared to isoproterenol control group (ISG), while isoproterenol control group (ISG) and *per se* (PSG) were compared to normal control group (NCG), ^a^ *p* < 0.001, ^ns^ *p* > 0.05 when compared to normal control group (NCG), ^b^ *p* < 0.05, ^c^ *p* < 0.01, ^d^ *p* < 0.001 when compared to isoproterenol control group (ISG). Total cholesterol (TC), triglyceride (TG), high-density lipoprotein (HDL), low-density lipoprotein (LDL), and very low-density lipoprotein (VLDL).

The lipid parameter viz. free fatty acid, triglycerides (TG), total cholesterol (TC), low-density lipoprotein (LDL), high-density lipoprotein (HDL), very low-density lipoprotein (VLDL) and phospholipids in the serum while TC, TG, free fatty acid and phospholipids was estimated in heart tissue. The result showed that there was an increase in lipid content while HDL level was reduced in the ISO group in serum and heart. PSG group did not show any significant changes in lipid content. The result was reversed in sericin and standard treated groups (SG1, SG2, and STG) proving the lipid-lowering potential of sericin (Figure 13).

### 3.10. Lysosomal Hydrolases

Lysosomal hydrolases enzymes play a substantial role in the inflammatory process. Isoproterenol prompted MI results in enriched lysosomal hydrolases activity which is held responsible for tissue damage and infarcted heart. It is postulated that if the myocardial tissue or cell membranes have become stable, primarily the lysosomal membranes, it may protract the overall life span of ischemic cardiac tissue and helps in preventing MI.

The enzyme lysosomal hydrolases were assessed and the results advocate that the enzymes Cathepsin-D, Cathepsin-B, β–Galactosidase, α–Galactosidase, and β–Glucosidase showed a highly significant (*p* < 0.001) increase in isoproterenol group (ISG) when compared to NCG. The standard group (STG) showed a statistically highly significant (*p* < 0.001) decrease in the level of α–Galactosidase, β–Galactosidase, β–Glucosidase, Cathepsin–B, Cathepsin-D when compared with isoproterenol group (ISG). In sericin group 1 (SG1) the level of β–Galactosidase, α–Galactosidase, Cathepsin-D, β–Glucosidase was found to be statistically very significantly (*p* < 0.01) whilethe level of Cathepsin–B was statistically significantly (*p* < 0.001) decreased when compared with isoproterenol group (ISG). In sericin group 2 (SG2) the level of Cathepsin–D, Cathepsin-B, β–Galactosidase and α–Galactosidase was found to be statistically very significantly (p < 0.01) while the level of β–Glucosidase was statistically highly significantly (*p* < 0.001) declined when it was matched with isoproterenol group (ISG).

**Figure 14:**
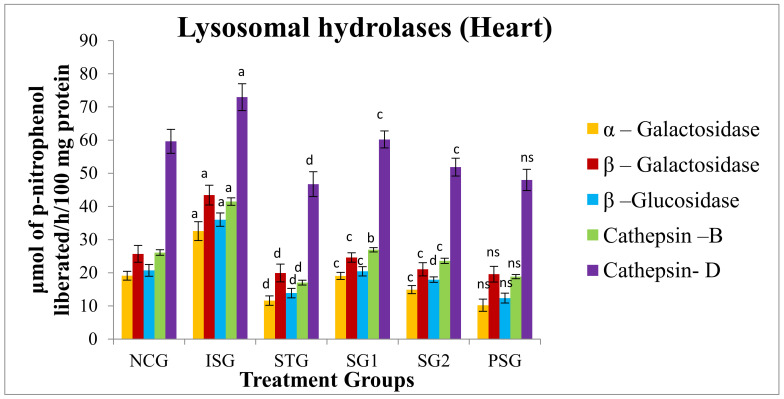
Lysosomal hydrolases level (Heart) of various treatment groups. Where, values are indicated as mean ± SD; (*n* = 6) in each group. Data were subjected to one-way ANOVA followed by Dunnett’s test when STG, SG1, SG2 group were compared to isoproterenol control group (ISG), while isoproterenol control group (ISG) and *per se* (PSG) were compared to normal control group (NCG), ^a^ *p* < 0.001, ^ns^ *p* > 0.05 when compared to normal control group (NCG), ^b^ *p* < 0.05, ^c^ *p* < 0.01, ^d^ *p* < 0.001 when compared to isoproterenol control group (ISG).

In the *per se* group (PSG) exposure of rats to sericin alone did not produce significant alterations (*p* > 0.05) in the level of tissue β–Glucosidase, α–Galactosidase, Cathepsin–D, Cathepsin-B and β–Galactosidase level in comparison with animals of the normal control group (NCG) (Figure 14).

### 3.11. Heart Mitochondrial Enzymes

Various different biochemical enzymes exist in mitochondria, for instance, α-Ketoglutarate dehydrogenase (KDH), Isocitrate dehydrogenase (IDH), Succinate dehydrogenase (SDH), and Malate dehydrogenase (MDH) which were measured in the various treated heart groups. Mitochondrial enzymes present in the heart were estimated and the isoproterenol group (ISG) showed statistically highly significant (*p* < 0.001) reduction in the level of MDH, KDH, IDH, and SDH when compared with the normal control group (NCG). The standard group (STG) showed a statistical highly significant (*p* < 0.001) increase in the level of MDH, KDH, IDH and SDH when compared witthe h isoproterenol group (ISG). In sericin group 1 (SG1) the level of MDH, KDH, and SDH were statistically very significantly (*p* < 0.01) while the level of IDH was statistically significantly (*p* < 0.05) increased when compared with the isoproterenol group (ISG). In sericin Group 2 (SG2) the level of KDH, IDH were statistically very significantly (*p* < 0.01), MDH was statistically highly significantly (*p* < 0.001) while the level of SDH was statistically significantly (*p* < 0.05) increased when compared with isoproterenol group (ISG).

**Figure 15:**
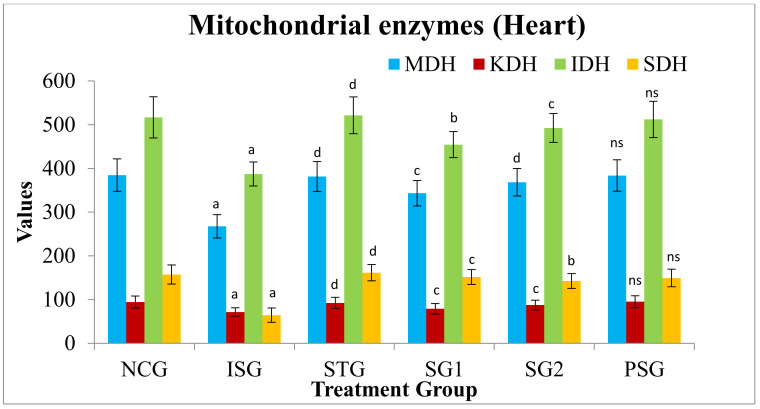
Mitochondrial enzyme level (heart) of different treatment groups. Where, values are indicated as mean ± SD; (*n* = 6) in each group. Data were subjected to one-way ANOVA followed by Dunnett’s test when STG, SG1, SG2 group were compared to isoproterenol control group (ISG), while isoproterenol control group (ISG) and *per se* (PSG) were compared to normal control group (NCG), ^a^ *p* < 0.001, ^ns^ *p* > 0.05 when compared to normal control group (NCG), ^b^ *p* < 0.05, ^c^ *p* < 0.01, ^d^ *p* < 0.001 when compared to isoproterenol control group (ISG). Malate dehydrogenase (MDH), Alpha-ketoglutarate dehydrogenase (KDH), Isocitrate dehydrogenase (IDH), Succinate dehydrogenase (SDH).

In the *per se* group (PSG) exposure of rats to sericin alone did not produce alterations (*p* > 0.05) in the level of tissue MDH, IDH, KDH, and SDH when compared with animals of the normal control group (NCG) (Figure 15).

Unit of Succinate dehydrogenase: nmoles of succinate oxidized/mg/min protein, Malate dehydrogenase: n moles of NADH oxidized/mg/min protein; Isocitrate dehydrogenase: n moles of α-ketoglutarate formed/mg/hr protein; α-Ketoglutarate dehydrogenase: nmoles of ferrocyanide formed/hr/mg protein.

### 3.12. Apoptotic Marker

#### 3.12.1. Expression of α-and β-Myosin Heavy Chain (α, β-MHC)

**Figure 16:**
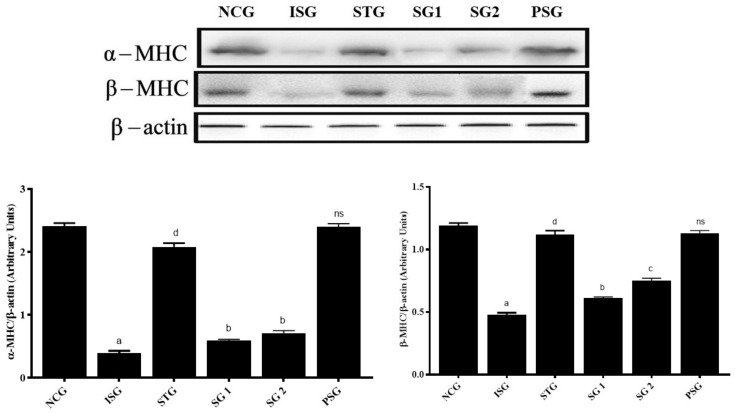
Western blotting analysis of α, β-MHC proteins: α, β-MHC proteins were estimated in tissue lysates by using western blot. β-actin was used as a reference protein. Graph displays different treatment groups vs. arbitrary value of α, β-MHC proteins. Data were subjected to one-way ANOVA followed by Dunnett’s test when STG, SG1, SG2 group were compared to isoproterenol control group (ISG), while isoproterenol control group (ISG) and *per se* (PSG) were compared to normal control group (NCG), ^a^ *p* < 0.001, ^ns^ *p* > 0.05 when compared to normal control group (NCG), ^b^ *p* < 0.05, ^c^ *p* < 0.01, ^d^ *p* < 0.001 when compared to isoproterenol control group (ISG).

The effect of sericin in isoproterenol-treated rats was supported by performing a western blotting analysis and results suggest that the expression of the α-myosin heavy chain (α-MHC) and β-myosin heavy chain (β-MHC) which is the major myofibrillar protein was highly significantly (*p* < 0.001) reduced in isoproterenol treatment groups (ISG), thus Isoproterenol attenuated the level of α-MHC and β-MHC in rat heart, while the level in *per se* group (PSG) was non-significant (*p* > 0.05) when compared to the normal control group (NCG), while the level of α-MHC and β-MHC in the standard group (STG) showed highly significant (*p* < 0.001), while the level of α-MHC in sericin treated group (SG1 and SG2) showed significant (*p* < 0.05) increase and the level of β-MHC in SG1 showed significant (*p* < 0.05) and SG2 group showed very significant (*p* < 0.01) increase when compared to isoproterenol group (ISG). The increase in myosin heavy chain (MHC) expression is more significant in SG2 than SG1 of sericin treated group when compared to the isoproterenol group (*p* < 0.05) (Figure 16).

#### 3.12.2. Flow Cytometry

**Figure 17:**
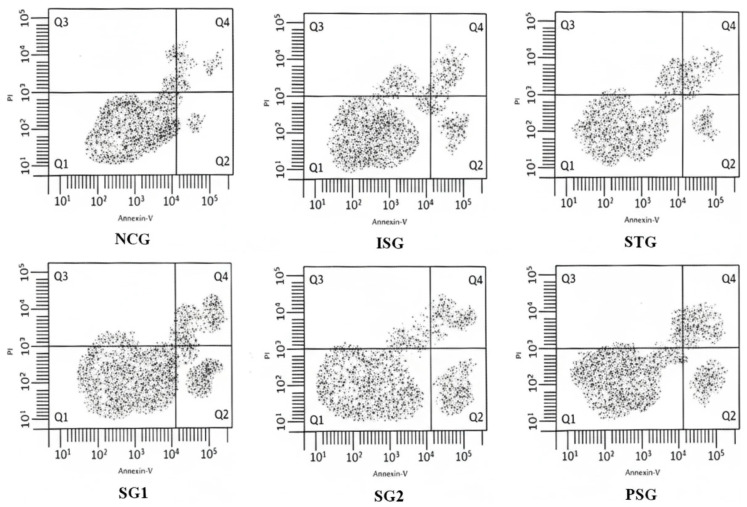
Cell necrosis/apoptosis finding using flow cytometry (Annexin V/FITC). The right upper quadrant (Annexin V+/PI+) represents early apoptotic cells and the right lower quadrant (Annexin V+/PI−) represents late apoptotic cells, the lower left quadrant (Annexin V−/PI) represents healthy cells and the left upper quadrant (Annexin V−/PI+) represents necrotic cells. Where FITC- Fluorescein isothiocyanate; PI-Propidium iodide.

Flow cytometry was performed to determine the apoptosis induced by Isoprenaline, sericin, and metoprolol. The flow cytometry data demonstrated that treatment with ISO resulted in a significant increase in necrotic, early, and late cell death (apoptosis) followed by a reduction in viable cells, and this impact changed into markedly decreased following sericin pretreatment with an increment in living cells and decreased rate of apoptosis. These findings proposed that ISO-triggered peculiar energy metabolism in the long run causes cell damage, and the sericin pretreatment reinstated the metabolism of energy and reduced tissue necrosis. (Figure 17).

### 3.13. Total Myocardial Collagen Content and Fibrosis Estimation

**Figure 18:**
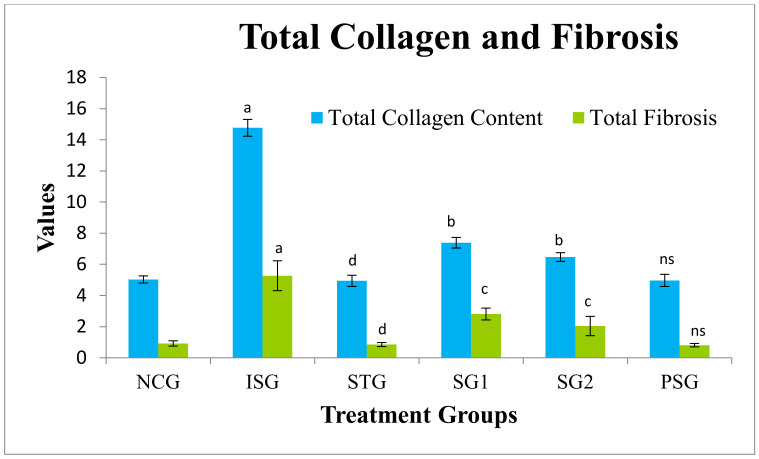
Total collagen and total fibrosis in different treatment groups where, values are expressed as mean ± SD; (*n* = 6) in each group. Data were subjected to one-way ANOVA followed by Dunnett’s test when STG, SG1, SG2 group were compared to isoproterenol control group (ISG), while isoproterenol control group (ISG) and *per se* (PSG) were compared to normal control group (NCG), ^a^ *p* < 0.001, ^ns^ *p* > 0.05 when compared to normal control group (NCG), ^b^ *p* < 0.05, ^c^ *p* < 0.01, ^d^ *p* < 0.001 when compared to isoproterenol control group (ISG). Where unit of collagen is mentioned as mg/gm while fibrosis is percentage (%).

Total myocardial collagen content and fibrosis were estimated and were found to be statistically highly significant in isoproterenol group rats (ISG). The sericin and standard treated group (SG1, SG2, and STD) showed a statistically very significant decrease in collagen content and fibrosis. No significant changes in the level of collagen content and fibrosis were observed in the PSG group. The result suggests that sericin reduces the synthesis of collagen in myocardiocytes, thus reducing the incidence of fibrosis, reduction of fibrosis and synthesis of collagens contributes the o protective effect of sericin against hypertrophy (Figure 18).

### 3.14. Cell (Myocyte) Size Determination

**Figure 19:**
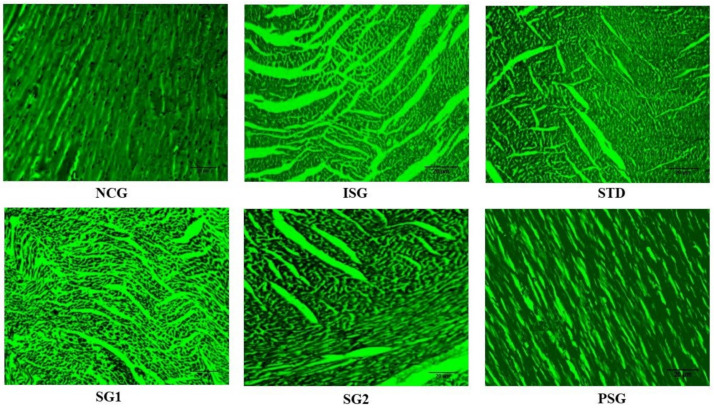
The images of myocyte cross-sectional area at 10× using cromogreen fusion dye of different treatment groups. The scaler bar has been mentioned in all images as 20 µm.

**Figure 20:**
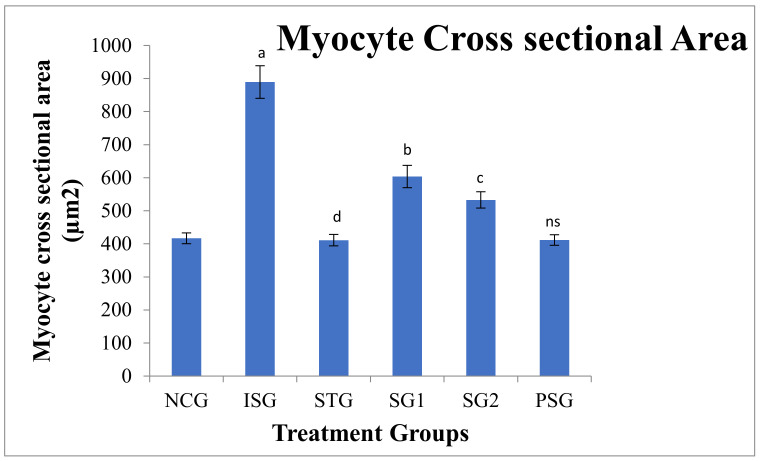
Myocyte cross-sectional area of different treatment groups. Where, values are expressed as mean ± SD; (*n* = 6) in each group. Data were subjected to one-way ANOVA followed by Dunnett’s test when STG, SG1, SG2 group were compared to isoproterenol control group (ISG), while isoproterenol control group (ISG) and *per se* (PSG) were compared to normal control group (NCG), ^a^ *p* < 0.001, ^ns^ *p* > 0.05 when compared to normal control group (NCG), ^b^ *p* < 0.05, ^c^ *p* < 0.01, ^d^ *p* < 0.001 when compared to isoproterenol control group (ISG).

The histopathology of the cardiac tissue was performed subsequently staining with cromogreen fusion dye in the different treatment groups later it was imperiled to image J software and determination of cell size was performed (Figure 19 and Figure 20).

The myocyte size was observed to be statistically highly significant (*p* < 0.001) in isoproterenol group rats (ISG) when compared to the normal control group (NCG). The sericin-treated group (SG1 and SG2) showed a statistically significant (*p* < 0.05) and statistically very significant (*p* < 0.01) decrease in myocyte size when compared to the isoproterenol group (ISG) respectively while standard (STG) showed statistically highly significant (*p* < 0.001) reduction in myocyte size when compared to Isoproterenol group (ISG). Myocyte size in (PSG) displayed no significant (*p* > 0.05) difference when compared to (NCG).

### 3.15. Histopathology

**Figure 21:**
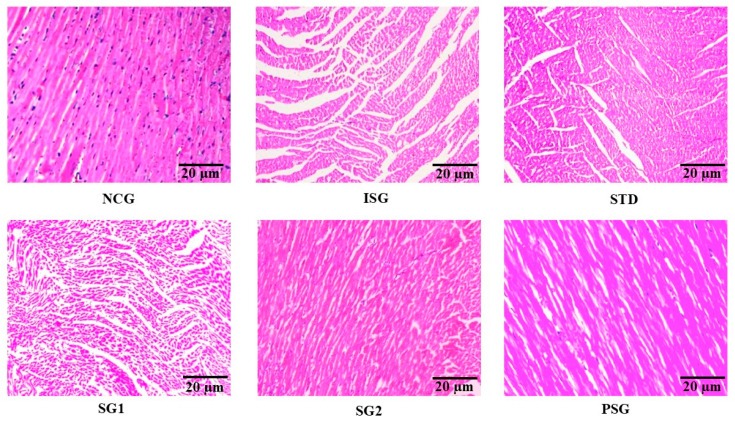
Different treatment groups displaying histopathological section of the heart. H&E stain of heart section of different treatment groups with scaler bar as 20 µm.

The histopathological end result showed that normal control rats administered every day with saline have confirmed the regular alignment of myocardium, endocardium, and epicardium in addition to papillary muscle fibres and vasculature in comparison to isoproterenol (ISG) group which displayed massive infarction with wall lesions thrombi and random acute aneurysms. The remaining treatment groups were in comparison to the isoproterenol (ISG) group, wherein the standard group confirmed regular intact myocardial tissue without infiltration, whilst the sericin group 1 (SG1) group confirmed focal lesions without an indication of myonecrosis, myophagocytosis, and lymphocytic infiltration. The sericin group (SG2) confirmed no loss of muscle fiber and shortage of vacuolated cells in conjunction with moderate edema and few inflammatory cells. The *per se* (PSG) showed regular myocardial tissue structure, a systemized pattern showing no vacuolation and myofiber striae with nuclei in center (Figure 21).

## 4. Discussion

Since time immemorial, natural origin medicines have played crucial roles in recent drug development, mainly for antibacterial and antitumor agents. However, insects have more than twofold the total number of species and in numerous parts of the world, entomotherapy has been practiced for as long as and frequently in combination with medicinal plants and is a significant alternative to allopathic medicine [55]. However, public observation of insects should not prevent scientific pioneers from advancing these mainly untapped resources. Certainly, if given the appropriate attention, insect-derived substances tend to provide more options for the future of natural origin product drug discovery [56].

By the end of the century, cardiovascular diseases (CVDs) have turned out to be the leading reason for death in India. Compared with people of European descent, CVD affects Indians at least a decade earlier and in their more productive age. For example, in western people only 23% of CVD deaths happen before the age of 70; in India, this figure is 52%. The World Health Organization (WHO) estimates that, with the current burden of CVD, India will lose $ 237 billion from productivity loss and spending on health care over 10 years (2005–2015) [57].

The grading of the heart was analysed and marks showed that the sericin and standard group (SG1, SG2 and STG) showed a decrease in edema, dilation of capillary, scar formation, and yellowing of the heart when compared with the isoproterenol group (ISG). These results may be due to the anti-inflammatory and injury curative properties of sericin.

Organ weight was assumed to be one of the sensitive markers of a test material effect, as countless significant variances in organ weight between treated and untreated (controlled) animals can appear in the absence of any kind of morphological changes [58]. The sericin group (SG1 and SG2) and standard group (STG) showed a significant decrease in the ratio when compared with isoproterenol group (ISG). Henry and Stephens have reported that the polyphenols do not favour water creation in heart tissues of mice in the study of chronic psychosocial hypertension decline [59].

Gross morphology estimation of RV, LV, and IVS explain that heart wall weight relies on the ventricular wall, septum width, and the magnitudes of the heart, which imitates remodeling and hypertrophic changes in the heart [60]. Results recommended that sericin group (SG1 and SG2) and standard group (STG) showed its action on geometric dimensions of the heart which was reflected by a significant dose-dependent decline in LV, RV, and IVS mass when compared with isoproterenol group (ISG). Prevention of increase in LV, RV, and IVS mass along with the preservation of cardiac function by sericin is symptomatic of its therapeutic potential in the developmental stage of cardiac hypertrophy.

Myocardium contains a rich concentration of many enzymes and once metabolically injured, releases its content into the extracellular fluid (ECF). An enhanced action of marker enzymes in serum is because of the leakage of enzymes from myocardial tissue as an outcome of isoproterenol-induced necrosis [61] and the number of enzymes that is present in the serum is directly related to the total number of necrotic cells [62].

In sericin group (SG1 and SG2) animals, serum marker enzymes were found to be considerably decreased when compared to isoproterenol group (ISG) rats. Sericin could have decreased the necrotic injury by its anti-free radical properties and barred the seepage of enzymes (AST, ALT, LDH, ALP, CK, CK-MB) from the tissue [63].

In the current study, sericin-treated groups (SG1 and SG2) showed reduced secretion of troponin by stabilizing the cellular membrane thus reducing degradation of the myofibrils which leads to a reduction in the release of troponin in blood. Cardiovascular troponins (cTn) found in serum, in particular troponin I (cTnI), is commonly released after severe myocardial necrosis which can be identified in serum and is a ramification of other cardiovascular illnesses [64,65,66].

The increased free radicals formed can undergo a reaction with polyunsaturated fatty acids inside the cell membrane which leads to lipid peroxidation; what happens, in turn, causes an outcome that reveals increased free radical production [67]. Ref [68] reported that the level of TBARS, HP, and CD were significantly amplified in the heart and plasma of ISO-induced experimental rats (ISG). In our study, rats given ISO also showed an increase in the amount of lipid peroxidation products (LHP, CD, and TBARS) in plasma and in the heart. The sericin treatment group (SG1 and SG2) reduced the amount of lipid peroxidation when matched to the isoproterenol group (ISG). The contrary effect of lipid peroxidative of sericin in ISO-administered rats might be due to the decline in hydroxyl radical and superoxide anion formation.

Studies in humans have shown that there is an inverse relationship between plasma total vitamins E and C and cardiac ischemic mortality [69,70,71,72]. The sericin-pretreated groups (SG1 and SG2) and the standard group (STG) showed a significant increase in vitamin E and vitamin C levels in both plasma and tissue compared to rats in the isoproterenol-administered group (ISG). Ref [73] has reported that sericin maintains cellular oxidative-antioxidant balance by reducing local O_2_ concentration and producing phenoxy radicals to suppress free radical-mediated lipid peroxidation. In our study, ISO (ISG)-induced GSH levels in rats decreased. The reduced GSH level could result in reduced free radical uptake, and these radicals might bring a variety of hostile myocardial reactions [72]. An increased GSH level was observed in rats treated with sericin groups (SG1 and SG2), which reduces free radical formation during myocardial injury.

Free radical rummaging enzymes, such as CAT, GR, GPx, SOD, and GST are the first line of cellular defense against oxidative stress [74]. The generation of antioxidant enzymatic defense system is a very significant reason to counteract the oxygen free radical facilitated tissue damage [75].

GPx also plays an important part in quenching H_2_O_2_ and other peroxides which if not lead to the creation of peroxyl and hydroxyl radicals. GR is an additional vital enzyme for preserving reduced glutathione intracellular level (GSH). As an important substrate for antioxidant enzymes, glutathione-S-transferase (GST) shields cellular components from the destructive effects of peroxides and ROS formed during metabolism [76]. Catalase (CAT) is a significant enzyme in avoiding the development of hydroxyl radicals. Catalase is accountable for reducing H_2_O_2_ to H_2_O. All through the myocardial infarction, these enzymes are anatomically and functionally reduced by free radicals, with ensuing myocardial damage [77].

In isoproterenol group (ISG) rats the activity of heart tissue antioxidant enzymes, was found to be decreased when compared to normal control group (NCG) rats. A similar result has been reported by [78,79,80,81,82,83]. Sericin groups (SG1 and SG2) rats revealed a marked surge in these parameters when equated to isoproterenol group (ISG) rats. Ref [84] has reported that sericin brings about its antioxidant part at several levels of the oxidative stages by preserving cellular oxidant and antioxidant equilibrium. Because sericin possesses antioxidant activity, in the contemporary study, it might have been credited for the preservation of the internal antioxidant equilibrium against isoproterenol facilitated cellular oxidation [84].

Decreased levels of serum total proteins, globulin, albumin, and A/G ratio were noticed in the ISO-induced group (ISG). A reduction in serum total proteins, albumin, globulin, and A/G ratio may be due to augmented free radical generation by the ISO administration, as protein sulfhydryl’s behave as a sacrificial antioxidant and stops the plasma lipid peroxidation, in addition, to being a target for oxidative damage. Pre-treatment with sericin (SG1 and SG2) significantly raised the levels of serum total proteins, albumin, globulin, and A/G ratio. This could be due to the ability of sericin to scavenge the noxious free radicals and constrain lipid peroxidation [85].

Clinical and experimental studies have suggested that rapid surges in serum glucose promote the infarct size [86], disturb the left ventricular function, and also promote microvascular obstruction [87]. This could be due to hyperglycaemia surges mediated by interstitial fibrosis and increases in the myocyte apoptosis that amplify left ventricular remodeling [88,89]. The glucose level was seen to increase in the isoproterenol treated group (ISG) while pre-treatment with sericin in SG1 and SG2 group have resulted in a very significant reduction in glucose levels.

The increment in the levels of cholesterol is responsible for an increment in the membrane fluidity, and alters internal viscosity, the internal chemical composition, and also regulates membrane penetrability [90]. The reason behind the increase in LDL in the blood is because of hyperlipidemia triggered by ISO along with a reduction in the action of lipoprotein lipase, which is responsible for the build-up of harmful accumulation in the arteries that may be responsible for causing coronary heart diseases (CHD) [91]. The findings of the current investigations indicate that the prior administration of sericin (SG1, SG2) significantly prevented the isoproterenol-treated degradation of membrane phospholipids and reduced serum TG, TC, LDL, free fatty acid, and elevated HDL while in heart tissue there was a lowering of TC, TG, phospholipids and free fatty acid in comparison to ISO group.

The deprivation of the myocardial membrane perceived in the present study could be due to improved efflux of lysosomal hydrolases enzymes into the cytosol from the sealed sacs [92]. Therefore, pretreatment with sericin (SG1, SG2) significantly increased levels of hydrolytic enzymes in the heart, justifying sericin’s mechanism of action as it also possesses antioxidant potential, which may be a key factor in maintaining the integrity of the lysosomal membranes, which prevent the release of the enzyme hydrolase into the circulation.

Mitochondria are significant subcellular organelles that are involved in the processing of energy and are vulnerable to oxidative stress [93]. In the present study, the actions of TCA cycle enzymes were decreased in the ISO-administered group (ISG). The decreased activities of TCA cycle enzymes which are situated on the superficial membrane of mitochondria and this could be exaggerated by the excessive creation of free radicals by ISO [94].

In the present study, the effects of the TCA cycle enzyme or mitochondrial respiratory chain enzymes such as, ICDH, α-KGDH, SDH and MDH were significantly decreased in ISO administered group (ISG) of rats when related to normal control group (NCG) rats. Pre-treatment with sericin in (SG1 and SG2) group rats significantly increased the level of enzyme when compared to the isoproterenol group (ISG). Inhibition of these enzyme’s actions by ROS may influence the mitochondrial substrate oxidation and catabolism of cellular energy [95].

The proteins within the muscle fibre that form the myofibril system are collectively known as myofibrillar proteins [96,97].

Myosin is an example of a molecular motor—a protein that results in muscular contraction and elasticity [98]. Myosin combines with actin to form a complex, the main part of the thin thread. Myosin heavy chain (MHC) comprises two isoforms i.e., α and β- MHC [99]. Relative expression tiers of α-and β-myosin heavy chain isoforms receive altered in sickness states along with cardiac hypertrophy and cardiac failure [49]. Exposure to isoproterenol in excessive doses results in deprivation of MHC which is the main myofibrillar protein. Few studies have additionally drawn interest in the direction of decreased myosin heavy chain (MHC) below various muscle losing situations [100,101]. Sericin (SG1 and SG2) however, conserved the MHC composition of cardiac tissue which is evident inside the manifestation of protein MHC in western blot results.

Annexin V/PI staining in flow cytometry analysis is based on the ability of the Annexin V protein to bind to phosphatidylserine (PS), which shifts from the inner membrane in the viable cells to the outer cell membrane upon initiation of apoptosis so it becomes accessible for Annexin V binding. The addition of PI enabled necrotic, viable, late apoptotic and early apoptotic cells to be distinguished [102]. The result suggested that the ISO group showed maximum apoptosis and reduced viable cells while these effects were reversed after pre-treatment with sericin through downregulation of caspase 3 and intracellular ROS generation while the increase in mitochondrial membrane potential [103].

Collagen fibres of the coronary heart are a network among myocytes, subsequently keeping the shape of the ventricles and spreading the contractile pressure from all cells of the myocardium to the ventricular lumen. The amino acid, hydroxyproline is a posttranslational end result of proline hydroxylation, and hydroxyproline is thought to be solely associated with collagen [104]. Collagen breakdown is most commonly related to the quantitative measurement of hydroxyproline concentration in the plasma, urine, and body tissue. Therefore, estimation of hydroxyproline gives valuable data for the diagnosis and forecast of diseases caused by ailments of the collagen metabolism [105]. In the current study, rats treated with sericin (SG1 and SG2) inverted isoproterenol-induced hypertrophic growth of the myocardium markedly thus decreasing the collagen accumulation and fibrosis formation considerably in the heart. A decrease in hydroxyproline level resulted after administration of sericin further established that sericin could lessen the isoproterenol-induced accumulation of collagen.

Cardiac fibrosis is a pathological situation with interstitial fibroblasts propagation and too much deposition of interstitial myocardial collagens leading to abnormal cardiac function [106], which would eventually advance into heart failure. So, reversal or inhibition of cardiac fibrosis may be a valuable strategy for the therapy of severe heart diseases [107].

This necrosis is followed by amplified fibrosis and hypertrophy. Hypertrophy has been considered a compensatory reaction to myocyte loss [108]. Thus, at this moment, it is not likely to completely rule out loss of myocyte as a key factor in any case of enlarged myocardial fibrosis or amplified collagen deposition (with respect to increased collagen content, which is proportional to the various stage of amplified hypertrophy) [54]. The results of this study demonstrate that fibrosis was decreased in sericin treated group (SG1 and SG2) due to a reduction in deposition of collagen and inhibiting activation of the adrenergic system. The result of this study indicates that this defense of myocardial necrosis could have been because of the antioxidant actions of sericin.

The cardioprotective study indicates that prior administration of sericin is effective in minimizing all the deleterious effects induced by isoproterenol, thereby justifying its use as a potent cardioprotective agent. The overall cardioprotective effect of sericin is probably attributed to many factors which were explored in this study, mainly its antioxidant property evidenced by its ability to reduce lipid peroxidation and to maintain the activities of free radical enzymes and non-enzymatic antioxidants, its membrane-stabilszing action, and its hypolipidemic property.

## 5. Conclusions

As per the data composed in this study, it could be specified that the administration of sericin orally at a dose of 500 mg/kg and 1000 mg/kg displayed potent cardio protective properties against isoproterenol induced experimental myocardial infarction thus decreasing inflammatory reactions and oxidative stress that leads to improved myocardial activity and reduced cardiac damage after myocardial ischemia. The outcomes of the above study delivered a new lead in the field of drug discovery which one is from natural sources so it is safe and effective and the possible each day consumption of sericin might be considered a cardioprotective drug for human beings in the future and this study also provides new insights into the development of different formulations of sericin which can act as targeted preventive therapies for cardiovascular diseases.

It can be considered a cardioprotective drug for humans, and this study also provides new insights into the development of different formulations of sericin that may act as specific preventive therapies for cardiovascular diseases. The results of the above study provided a new lead in the field of drug discovery that is derived from natural sources, so it is safe and effective, and the possible daily intake of Sericin could be considered a cardioprotective drug for humans in the future.

The future prospective of this work is to plan and conduct a clinical trial on sericin so that we can move toward getting a new potent, safe, cost-effective natural drug for the treatment of these cardiac ailments. The sericin can be used as an ingredient in the development of various formulations which can be used in the treatment of myocardial infarction. Novel drug delivery formulations such as nanoformulations and liposomes can also be prepared by using sericin as a key active pharmaceutical ingredient, thus exploring more avenues for the improvement of drug delivery and drug targeting.

## Figures and Tables

**Table 1:** Experimental protocol displaying details of groups, treatment its route, dose, and duration.

Groups	Treatment Provided	Dose, Route and Duration
Normal Control Group (NCG)	Rats were administered with distilled water	2 mL/kg/day/p.o. for continuous 28 days
Isoproterenol Control Group (ISG)	Rats were administered with Isoproterenol	85 mg/kg/s.c./once a day administered on the 29th and 30th days.
Standard Group (STG)	Rats were administered with Metoprolol + Isoproterenol	10 mg/kg/day/p.o. for continuous 28 days + 85 mg/kg/s.c. once a day administered on the 29th and 30th days
Sericin Group 1 (SG1)	Rats were administered with Sericin + Isoproterenol	500 mg/kg/day/p.o. for continuous 28 days + 85 mg/kg/s.c. once a day administered on the 29th and 30th days
Sericin Group 2 (SG2)	Rats were administered with Sericin + Isoproterenol	1000 mg/kg/day/p.o. for continuous 28 days + 85 mg/kg/s.c. once a day administered on 29th and 30th day
*Per se* Group (PSG)	Rats were administered with Sericin alone	1000 mg/kg/day/p.o. administered continuously for 28 days

**Table 2:** Grading of gross examination of rat heart.

Grade No.	Characteristics
Grade-0	No lesion
Grade-1	Inflammation and redness, capillary dilatation
Grade-2	Edema, capillary dilatation, ventricle portion yellowish
Grade-3	Scar formation, yellowish color of atrium and ventricle part of heart
Grade-4	Diffuse heart, scar formation, and yellowish color of atrium and ventricle

**Table 3:** Different treatments group showing various grades of heart.

Treatment Groups	Grade
Normal Control (NCG)	Grade 0
Isoproterenol Control Group (ISG)	Grade 4
Standard Group (STG)	Grade 1
Sericin Group 1(SG1)	Grade 2
Sericin Group 2(SG2)	Grade 1
*Per se* Group (PSG)	Grade 0

**Table 4:** Existence of troponin-I in the different experimental groups, where −ve represents the absence of troponin, while +ve represents the presence of troponin.

Treatment Group	Number of Animals					
	1	2	3	4	5	6
NCG	−ve	−ve	−ve	−ve	−ve	−ve
ISG	+ve	+ve	+ve	+ve	+ve	+ve
STG	−ve	−ve	−ve	−ve	−ve	−ve
SG1	−ve	+ve	−ve	+ve	+ve	−ve
SG2	+ve	+ve	−ve	−ve	−ve	−ve
PSG	−ve	−ve	−ve	−ve	−ve	−ve

## Data Availability

Not applicable.

## Abbreviations

SOD	Superoxide dismutase
CAT	Catalase
GPX	Glutathione peroxidase
GR	Glutathione reductase
GST	Glutathione S-Transferase
IDH	Isocitrate dehydrogenase
MDH	Malate Dehydrogenase
SDH	Succinate dehydrogenase
α-KGDH	α-ketoglutarate dehydrogenase
TP	Total Protein
AL	Albumin
GL	Globulin
A/G ratio	Albumin/globulin ratio
AST	Aspartate aminotransferase
ALT	Alanine aminotransferase
LDH	Lactate dehydrogenase
CK	Creatine kinase
CK-MB	Creatine kinase myocardial band
ALP	Alkaline phosphatase
TBARS	Thiobarbituric acid reactive substances
GC	Glucose
TC	Total cholesterol
TG	Triglyceride
HDL	High density lipoprotein
LDL	Low density lipoprotein
VLDL	Very low density lipoprotein
CVD	cardiovascular diseases
CHD	Coronary heart disease
IHD	Ischemic heart disease
SPF	Specific pathogen-free
CDRI	Central drug research institute
CPCSEA	Committee for the purpose of control and supervision of experiments on animals
IAEC	Institutional animal ethical committee
p.o.	Per oral
b.w.	Body weight
*w*/*v*	weight/volume
LV	Left ventricle
RV	Right ventricle
IVS	Intra ventricular septum
RIPA	Radioimmunoprecipitation assay
Tris-HCl	Tris(hydroxymethyl)aminomethane hydrochloride
NaCl	Sodium chloride
EGTA	Ethylene glycol-bis(β-aminoethyl ether)-N,N,N′,N′-tetraacetic acid
Na_2_EDTA	Ethylenediaminetetraacetic acid disodium salt dihydrate
Na_3_VO_4_	Sodium orthovanadate
PMSF	Phenylmethylsulfonyl fluoride
SDS–PAGE	Sodium dodecyl sulphate–polyacrylamide
PVDF	Polyvinylidene fluoride
TBST	Tris-buffered saline and Polysorbate 20
ECL	Enhanced chemiluminescence
FITC	Fluorescein isothiocyanate
PBS	Phosphate buffered saline
PI	Propidium iodide
FACScan	Fluorescence activated cell analyzer
THP	Total hydroxyproline content
CF	Conversion factor
H&E	Hematoxylin and eosin
NIH	National institutes of health
NCG	Normal control group
PSG	Per se group
ISG	Isoprenaline control group
STG	Standard treated group
SG2	Sericin group 2
SG1	Sericin group 1
MHC	Myosin heavy chain
ECF	Extra cellular fluid
cTn	cardiovascular troponins
cTnI	Cardiovascular troponin I
HP	Hydroperoxides
CD	Conjugated dienes
ROS	Reactive oxygen species
ISO	Isoprenaline (Isoproterenol)
MI	Myocardial Infarction
TCA	Tricarboxylic acid
PS	Phosphatidylserine
ECM	Extra cellular matrix
