# Correction: Ahsan et al. Effectual Endeavors of Silk Protein Sericin against Isoproterenol Induced Cardiac Toxicity and Hypertrophy in Wistar Rats. *Life* 2022, *12*, 1063

**DOI:** 10.3390/life15101607

**Published:** 2025-10-16

**Authors:** Farogh Ahsan, Tarique Mahmood, Tanveer A. Wani, Seema Zargar, Mohammed Haris Siddiqui, Shazia Usmani, Arshiya Shamim, Muhammad Wahajuddin

**Affiliations:** 1 Department of Pharmacy, Integral University, Dasauli, Kursi Road, Lucknow 226026, India; faroghlab@gmail.com (F.A.); shazia@iul.ac.in (S.U.); arshiyanadeemsiddiqui@gmail.com (A.S.); 2 Department of Pharmaceutical Chemistry, College of Pharmacy, King Saud University, P.O. Box 2457, Riyadh 11451, Saudi Arabia; twani@ksu.edu.sa; 3 Department of Biochemistry, College of Science, King Saud University, P.O. Box 22452, Riyadh 11451, Saudi Arabia; szargar@ksu.edu.sa; 4 Department of Bioengineering, Integral University, Dasauli, Kursi Road, Lucknow 226026, India; mohdharis.siddiqui@gmail.com; 5 Institute of Cancer Therapeutics, School of Pharmacy and Medical Sciences, Faculty of Life Sciences, University of Bradford, Richmond Road, Bradford BD7 1DP, UK; m.wahajuddin@bradford.ac.uk

## Error in Figure

In the original publication [1], there was a mistake in Figure 21 as published. The wrong image was inadvertently included as Figure 21 in our published paper. We regret this mistake and sincerely apologize for any confusion it may have caused.

To address this issue, we are attaching:

1. The original images of each group from the correct experimental dataset.
2. The reformatted and corrected version of Figure 21 which should replace the incorrect figure currently in the manuscript. The corrected Revised Figure 21 appears below.

The authors state that the scientific conclusions are unaffected. This correction was approved by the Academic Editor. The original publication has also been updated.

**Figure 21:**
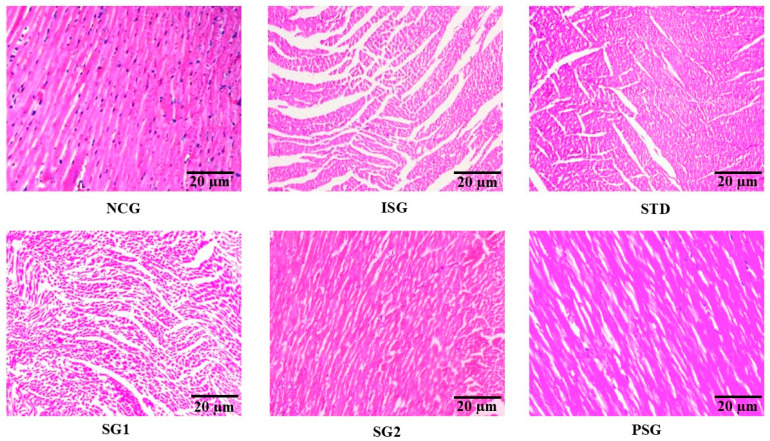
Different treatment groups displaying histopathological section of the heart. H&E stain of heart section of different treatment groups with scaler bar as 20 µm.
